# Pathogenesis of Renal Disease in Systemic Lupus Erythematosus—The Role of Autoantibodies and Lymphocytes Subset Abnormalities

**DOI:** 10.3390/ijms16047917

**Published:** 2015-04-09

**Authors:** Desmond Y. H. Yap, Kar N. Lai

**Affiliations:** Nephrology Division, Department of Medicine, The University of Hong Kong, Hong Kong, China; E-Mail: drdesmondyap@gmail.com

**Keywords:** autoantibodies, lupus nephritis, lymphocytes, pathogenesis, subsets

## Abstract

Lupus nephritis (LN) is a common and severe organ manifestation of systemic lupus erythematosus (SLE), and is associated with significant patient morbidity and mortality. Autoantibodies and aberrations in lymphocyte subsets have putative roles in the pathogenesis of SLE and LN, and might reflect disease activity and are amenable to immunosuppressive treatments. Anti-DNA is one of the well-studied autoantibodies, which correlates with disease activity and has direct nephritogenic effects on resident renal cells and various glomerular components. Other important autoantibodies in the pathogenesis of LN include anti-C1q, anti-α-actinin and anti-nucleosome antibodies. Changes in naive and memory B cells and plasma cells have been observed in SLE and LN patients. These B cell subsets exert diverse effects during pathogenesis of LN such as production of autoantibodies, secretion of proinflammatory and anti-inflammatory cytokines and presentation of auto-antigens to effector cells. Aberration of T lymphocytes, especially the T-helper subsets, is also highly pertinent in the development of LN. In this context, important T helper subsets include Th1, Th2, Th9, Th17, T_Reg_ and follicular T-helper cells. The growing knowledge on these autoantibodies and lymphocyte subset abnormalities will enhance our understanding of SLE and LN, and hence help devise better strategies for disease monitoring and treatment.

## 1. Introduction

Systemic lupus erythematosus (SLE) is a chronic autoimmune disease with multi-systemic involvements. Lupus nephritis (LN) is a common and serious organ manifestation of SLE and is associated with substantial patient morbidity and mortality in certain racial groups such as Africo-Americans and Asians [1,2,3]. The pathogenesis of SLE and LN is highly complex. Genetic predispositions, proinflammatory and anti-inflammatory cytokines, autoantibodies, lymphocyte subset abnormalities as well as defects in the complement systems all have putative roles in the development of SLE. Among these factors, the contribution of autoantibodies and lymphocyte subset aberrations in the pathogenesis of renal disease in SLE cannot be over-emphasized. In this context, there is a close link between the production of autoantibodies and the abnormalities in lymphocyte subpopulations. In addition, changes in the level of autoantibodies and lymphocyte subsets can reflect disease activity in LN and are potential targets of immunosuppressive therapies. Hence, an enhanced understanding of these nephritogenic autoantibodies and lymphocyte subsets will help develop novel strategies for disease activity monitoring and treatment for LN.

## 2. The Role of Autoantibodies

The production of autoantibodies is one hallmark feature of SLE, and autoantibodies are frequently used as biomarkers for diagnosis and disease monitoring. While mounting evidence had suggested the pathogenic significance of conventional autoantibodies, new target auto-antigens and their relevant autoantibodies have been continually identified to help understand the pathogenesis of LN as well as to expand our diagnostic and disease monitoring tools. Various pathogenic mechanisms of these autoantibodies in LN have been proposed which include formation of circulating immune complexes which will deposit in renal tissues, binding of autoantibodies to “planted antigens” entrapped within the kidney parenchyma, and direct binding of autoantibodies to *in*-*situ* cross-reactive antigens on resident renal cells and extra-cellular matrix components. The following discussion highlights some of the autoantibodies that might have pathogenic significance in LN.

### 2.1. Anti-dsDNA Antibody

Anti-dsDNA antibody is an illustrative example of an autoantibody which has great significance in pathogenesis, diagnosis and disease activity monitoring in LN. The pathogenic role of anti-dsDNA is strongly supported by its close association with clinical disease activity [4], and its detection in eluates obtained from renal biopsy of LN patients [5,6,7]. Accumulating data have suggested that anti-dsDNA could directly bind to different resident renal cells and also the extracellular matrix components, and induce inflammation and cell function changes. In either pre-nephritic NZB/W F1 or BALB/c mice, the injection of anti-dsDNA antibodies would result in direct (cross-reactive) or indirect (chromatin-mediated) binding to mesangial cells [8,9,10]. Previous studies have also demonstrated that anti-dsDNA isolated from LN patients could bind to human mesangial cells and its binding activity correlated with disease activity [11]. An array of anti-dsDNA binding targets on mesangial cells has been proposed and they include annexin II, α-actinin, laminin or heparin sulphate [9,12,13,14]. In this context, the binding activity of anti-dsDNA to annexin II closely links with disease activity in human LN, and glomerular annexin II expression co-localizes with IgG and C3 deposits and correlates with severity of nephritis [9]. The relationship between anti-DNA and α-actinin is intriguing. Indeed, anti-α-actinin antibodies are detected in approximately one fifth of SLE patients [12]. One should also appreciate that more than 90% of patients with anti-dsDNA antibody had cross-reactivity to α-actinin [12]. Raised anti-α-actinin antibodies titres are detected prior to or at disease onset in LN patients when compared with active or inactive lupus patients who did not have evidence of nephritis [12]. Anti-α-actinin antibodies generated by Epstein–Barr virus transformation of lymphocytes isolated from SLE patients would cross-react with α-actinin and these cross-reacting antibodies could bind to mesangial cells and isolated glomeruli *ex vivo* [13]. Furthermore, alpha-actinin 4 and a splice variant of α-actinin 1 are both highly expressed in mesangial cells isolated from MRL/lpr mice and these observations suggested that upregulated α-actinin expression may affect the extent of immunoglobulin deposition in the pathogenesis of LN [14]. Nucleosomes are also important intra-renal targets of autoantibodies, and the loss of intra-renal nuclease would promote *in*-*situ* nucleosome accumulation and thus the development and binding of autoantibodies [15,16]. The presence of circulating chromatin fragments is important for glomerular mesangial matrix deposition of anti-dsDNA antibody-containing immune complexes in murine LN [10]. Also, the use of heparin to enhance degradation of nucleosomes could reduce their immunogenicity and prevent binding of nucleosome-IgG complexes in glomeruli of NZB/W F1 mice [17]. Anti-dsDNA isolated from LN patients could also bind to human proximal renal tubular epithelial cells (PTEC) and induce proinflammatory cytokine secretion and cell morphology alterations [18]. Affinity-purified autoantibodies to native DNA isolated from NZB/W F1 mice and two SLE patients with active LN exhibited cross-reactivity with the A and D SnRNP polypeptides and interaction with pig kidney cells [19]. Autoantibodies from one of these patients bind mostly to the cell surface and resulted in much significant complement-mediated cytolysis when compared to the patient whose autoantibodies were internalized [19]. It was also reported that murine anti-dsDNA antibody (mAb 3E10) had binding activity to human renal tubular cells, and residues required for binding DNA, but not HP8/HEVIN, were essential for antibody penetration [20]. Such observation implied that cellular penetration required the presence of DNA or antibody binding to a membrane component with close resemblance to DNA. These studies on how anti-dsDNA antibodies interact with PTEC have provided insight on the pathogenesis of tubulo-interstitial inflammation in LN. Circulating anti-endothelial cell antibodies have been detected in a substantial proportion of SLE patients, and correlates with disease activity [21,22]. Previous studies have reported that murine anti-dsDNA antibodies, in the presence of DNA, could bind to a 46 kDa plasma membrane protein on human umbilical vein endothelial cells (HUVEC), and the binding of DNA to HUVEC protein was augmented with IL-1α or TNF-α [23,24]. Also, anti-dsDNA binding to endothelial cells could stimulate release of IL-1 and IL-6, enhance von Willebrand factor cell surface expression, and upregulate IL-8, TGF-β and nitric oxide (NO) synthetase gene expression [25,26,27]. Previous studies have also shown that anti-dsDNA possessed *in vitro* and *in vivo* podocyte-binding activity [11,13,16]. Histopathological changes such as podocyte foot process effacement as well as subepithelial and subendothelial deposits were observed in BALB/c severe combined immunodeficiency (SCID) mice administered with a cell line that produced anti-dsDNA/α-actinin antibodies [13]. Although these findings implied α-actinin might be one candidate target of anti-dsDNA when they bound to endothelial cells, Mjelle *et al.* observed that anti-dsDNA with high affinity to α-actinin did not bind to such component in nephritic kidney sections, but rather to nucleosome-containing structures within the mesangial matrix or the glomerular basement membranes (GBM) [16]. The study by Krishnan *et al.* have also echoed that anti-dsDNA could bind directly to GBM and induce severe glomerulonephritis [28].

### 2.2. Anti-C1q Autoantibody and Other Autoantibodies

C1q is a component of C1 that belongs to the classical complement activation pathway. The binding of C1q to the Fc portion of IgG or IgM within immune complexes induces conformational changes in its collagen-like region to expose neoantigens, and facilitates the formation of autoantibodies against C1q [29,30,31]. Anti-C1q autoantibodies from active LN patients could prevent the clearance of apoptotic cells and interfere with the complement classical pathway activation *in vitro* [32]. Several studies have demonstrated that serum anti-C1q IgG titres correlated well with nephritic flares in SLE patients [33,34,35]. Although the exact pathogenic roles of anti-C1q in LN remained elusive, the good correlation with clinical activity and its association with complement activation pathways in LN strongly supported its role in development of LN. Other investigators have also reported on different autoantibodies and their pathogenic mechanisms in LN. One recent study suggested that circulating mesangial cell-binding IgG in proliferative LN patients correlated with serological activity and could predict renal flares [36]. Mesangial cell-binding IgG_1_, but not other subclasses, correlated with the amount of mesangial deposits on electron microscopy, and hence suggested a potential pathogenic significance of IgG_1_ in proliferative LN. Polyclonal IgG isolated from LN patients can decrease tyrosine phosphorylation of podocytic protein such as tubulin [37]. Other autoantibodies that have been reported to assume pathogenic potential in LN include anti-Ro, anti-Smith, anti-CRP, anti-serum amyloid protein and anti-ribosomal P protein antibodies [3,38,39]. The target antigens and the downstream cascade involved in the binding of these autoantibodies, however, remain to be elucidated.

## 3. The Role of B Lymphocytes and Its Subsets

**Figure 1:**
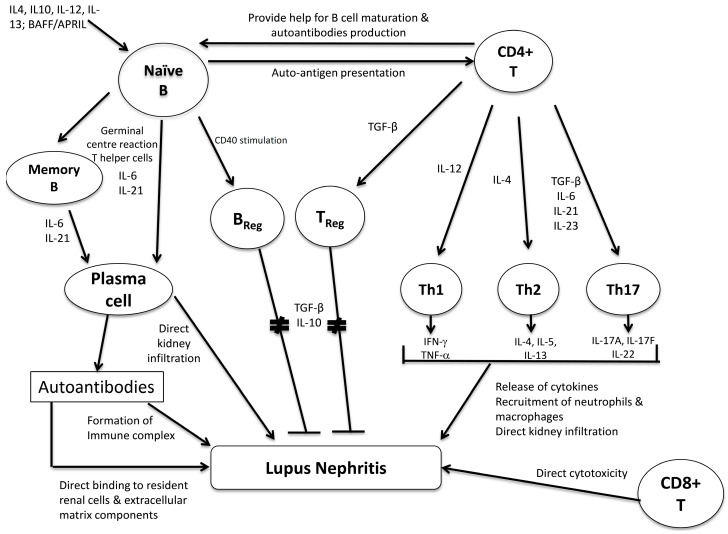
The interaction between B and T lymphocyte subsets and their possible pathogenic roles in lupus nephritis.

B lymphocytes have pleiotropic effects in the development of LN, including the generation of autoantibodies, secretion of proinflammatory and anti-inflammatory cytokines, auto-antigen presentation and direct infiltration to the kidneys (Figure 1). Antibody-secreting B cells and plasma cells were abundant in the kidneys of NZB/W F1 mice, and B cells that secrete nephritogenic anti-dsDNA could be isolated from nephritic MRL/lpr mice [40,41]. Interestingly, the frequency and autoantibody-secreting ability of the auto-reactive plasma cells within the kidney or bone marrow in NZB/W F1 mice were almost comparable, and the number of intra-renal plasma cells correlated with anti-dsDNA levels, and the activity and chronicity indices in LN [41]. Previous studies have reported that CXCL13 were highly expressed in NZB/W F mice with nephritis, and this would promote renal and other organ trafficking of B1 lymphocytes [42]. The pathogenic effect of B cells in LN is not limited to autoantibodies production. While genetically manipulated MRL/lpr mice that were incapable of antibody secretion still developed severe nephritis, B cell-deficient MRL/lpr mice were prevented from severe nephritis [43,44]. The improvement in renal manifestations in B cell-deficient mice might be explained by the failure of development of activated CD4^+^ and CD8^+^ T cells. IL-10-producing B cells, also known as regulatory B cells, also serve to regulate immune response and prevent autoimmunity [45]. However, the characterization of these IL-10-producing B cells and their roles in LN remains undefined. In human SLE, an increase in memory B cells and plasma cells coupled with a decrease of naive B cells are detected in the peripheral blood [46,47]. The antigen-experienced memory B cells have reduced regulation by FcγRIIb, and can be easily activated by the combination of toll-like receptor agonist or B cell activating factor (BAFF) [47,48]. Moreover, memory B cells have a low proliferation rate and are thus less susceptible to conventional immunosuppressive agents which are cell-cycle dependent, and hence a higher tendency to be involved in LN relapse [49]. In addition, SLE patients with active disease showed a marked expansion in plasma cells, and the frequency of circulating plasma cells correlated with disease activity scores, anti-dsDNA titre and serum immunoglobulin production [49,50]. In LN patients, local infiltration of B cells in the kidneys is associated with more severe renal disease and is augmented by BAFF [51]. The diverse and pivotal roles of B lymphocytes in the pathogenesis of SLE have aroused growing interest to use B cell-depleting therapy for the treatment of LN, but the human clinical trial data are less promising than one would have expected. In murine lupus models, the ablation of B cells via gene deletion or anti-CD20 administration prevents early death, inflammatory infiltrates and organ damage, while long-term administration of anti-CD20 eliminates autoantibody-secreting cells and is associated with reduction in survival niche for renal plasma cells [52,53]. However, recent randomized controlled trial data suggested that add-on rituximab (anti-CD20) to standard-of-care treatments did not improve clinical efficacy [54,55]. However, rituximab treatment might be useful in resistant LN or other refractory lupus manifestations [56]. Two randomized placebo-controlled phase III studies showed that belimumab (a BLys antagonist), when add-on to standard treatment, could enhance response rates in SLE patients without severe nephritis [57,58]. *Post hoc* analysis of patients with renal involvement from these studies suggested an efficacy signal of belimumab in LN, and hence further clinical trials are underway [59].

## 4. The Role of T lymphocytes and Its Subsets

T lymphocytes assume crucial roles in the development of LN such as provision of help to B cells to mature and produce autoantibodies, orchestration of B and T cell responses, release of proinflammatory and anti-inflammatory cytokines, and also the direct infiltration and cytotoxicity to renal parenchymal tissues (Figure 1). CD4^+^ helper T cells and CD8^+^ cytotoxic T cells are the two major subpopulations of T lymphocytes. Previous studies have revealed abundant mononuclear cells, CD4^+^ T cells and CD8^+^ T cells within the tubulo-interstitial lesions in LN patients [60]. However, depletion of CD8^+^ cytotoxic T cells in lupus-prone mice has produced conflicting data in SLE. β2-microglobulin deletion, which eliminated MHC Class I molecules and hence markedly impaired CD8^+^ T cell response, accelerated disease in NZB/W F1 mice [61]. Prolonged anti-CD8 administration also failed to alleviate disease in MRL/lpr or NZB/W F1 mice, in contrast to the favorable response to anti-CD4 treatment [62,63]. Studies on CD4^+^ helper T cells, however, have generated more important data regarding the development of SLE and LN. The following discussion highlights some of these important findings on CD4^+^ T cell subsets in the pathogenesis of LN.

### 4.1. T-Helper Cells

#### 4.1.1. Th1/Th2 Subsets

Th1 and Th2 responses represent two major T helper cell responses. In human LN, the Th1/Th2 balance exerts significant impact on the renal histopathological manifestations of LN. A predominant Th1 response was observed in both peripheral and renal tissues of patients with diffuse proliferative LN [64]. Okamoto *et al.* revealed that activated Th1 cells from kidneys of MRL/lpr mice with proliferative LN expressed high levels of T-cell receptor with IFN-γ, but not the cytokines relevant to Th2 or Th17 responses [65]. Urinary IL-12p70 elevation, which reflects a skewed Th1 response, is evident in diffuse proliferative but not membranous LN patients [66]. It was also demonstrated that peripheral IFN-γ/IL-4 ratio in diffuse proliferative LN was much higher than that in membranous LN, and was also correlated with the NIH activity index on renal biopsies [67,68]. MRL/lpr mice with deficiency of IFN-γ or its receptor had significantly milder proliferative LN changes [69,70]. In addition, IL-12-deficient MRL/lpr mice were associated with delay onset of diffuse proliferative LN and less severe renal histopathological changes [71]. Although these data have illustrated the importance of IFN-γ and Th1 response in SLE, it should be highlighted that IFN-γ might not be as important in the pathogenesis of human lupus as it is for the murine MRL/lpr model. On the other hand, Th2 response favored the development of membranous LN lesions in lupus-prone mice. MRL/lpr mice with WSX-1 defect exhibited pronounced Th2 but impaired Th1 responses, and developed renal lesions akin to human membranous LN [72]. As initial Th1 response is dependent on WSX-1, the loss of WSX-1 would polarize immunological functions towards Th2 response, and resulted in such phenotypic shift from diffuse proliferative to membranous LN in MRL/lpr mouse. These observations suggested the crucial role of Th2 response in the development of membranous LN.

#### 4.1.2. Th17 and T_Reg_

There is growing interest and data in Th17 and regulatory T cells (T_Reg_) on their pathogenic roles in various autoimmune diseases including SLE. Th17 is a recently identified T helper cell subset characterized by the production of its signature cytokine IL-17, and has been implicated in causing inflammation and tissue injury in autoimmune disorders. In NZB/W F1 mice, there is significant renal infiltration of Th17 cells accompanied with abundant IL-17 production [73]. CD3^+^CD4^−^CD8^−^ T lymphocytes from MRL/*lpr* mice express copious amount of IL-17 and that as disease progresses, the expression of IL-17 and of IL-23 receptor in lymphocytes from these mice increases [74]. Cells extracted from MRL/lpr mice lymph nodes, upon treatment *in vitro* with IL-23, induced nephritis when transferred to non-autoimmune lymphocyte-deficient Rag-1(−/−) mice, and significant immunoglobulin and complement deposition were detected in these recipient mice [74]. Furthermore, IL-23 receptor deficiency or treatment with anti-IL23 monoclonal antibody ameliorates the development of LN in lupus-prone mice [75,76]. These data all suggested that an aberrant IL-17/IL23 axis is pivotal in the pathogenesis of murine LN. In human SLE, CD3^+^CD4^−^C8^−^ T cells produced high levels of IL-17 and IFN-γ, underwent expansion when stimulated *in vitro* with an anti-CD3 Ab in the presence of accessory cells, and were detected in the renal biopsies of LN patients [77]. SLE patients showed a significantly higher percentage of Th17 cells when compared to healthy controls, and these Th17 cells showed high expression levels of co-stimulatory molecules CD80 and CD134 [78]. Infiltration of Th17 cells with high CD134 expression was also detected in kidneys of LN patients [78]. The increased frequencies of Th17 cells was also coupled with significantly higher IL-17 and IL-23 levels when compared to healthy controls, and the number of Th17 cells correlates with disease activity scores (total and renal systemic lupus erythematosus disease activity index (SLEDAI)) and histopathological changes in the kidney [79]. Recent works by Zickert *et al.* have shown that high baseline IL-17 level was predictive of an unfavorable histopathological response, and non-responders (according to British Isles Lupus Activity Group (BILAG) scores) showed high circulating IL-23 levels [80]. These results suggested that a subset of LN patients exhibited Th17 phenotype that might affect response to treatment and could be evaluated as a biomarker for poor therapeutic response. Nevertheless, some recent findings have produced conflicting results regarding the role of Th17 in LN. For instance, Schmidt *et al.* revealed low frequency of infiltrating CD3^+^IL-17A^+^ cells in the kidneys of MRL/lpr and NZB/W F1 mice [81]. Also, IL-17A deficiency did not influence the renal or histological parameters in MRL/lpr mice with LN, nor did IL-17A antagonism alter the clinical course in nephritic NZB/W F1 mice [81]. While most data on Th17 cells still favors a pathogenic role in SLE and LN, the exact roles and pathogenic mechanisms of Th17 in LN needs to be further characterized. T_Reg_ cells (CD4^+^CD25^+^FOXP3^+^) play a protective role in autoimmune disorders. The treatment of anti-mouse thymocyte globulin followed by TGF-β1 in MRL/lpr mice induced T_Reg_ differentiation, and inhibited progression of proteinuria and improved renal histopathologies [82]. In murine LN, depletion of T_Reg_ in NZB/W F1 accelerated the onset of diffuse proliferative LN whereas their transfer into CD4/CD25 knock-out mice retarded the development of nephritis [53]. In human LN patients, a substantial shrinkage of CD4^+^CD25^high^ and CD4^+^CD25^+^FoxP3^+^ T_Reg_ cells was observed in the peripheral blood, and such reduction in T_Reg_ number was accompanied with lower serum TGF-β1 levels [83] Moreover, the increase of peripheral Th17 cells was coupled with a decrease in T_Reg_ in LN patients, suggesting that the importance of Th17/T_Reg_ imbalance in the pathogenesis of renal disease in SLE [84].

#### 4.1.3. Other T Helper Subsets (Follicular T Helper Cells, Th9 and Th22 Cells)

Follicular T helper cells (T_FH_) are antigen-experienced CD4^+^ T cells found at the B cell follicles of secondary lymphoid organs, and are characterized by the surface marker expression of CXCR5. They serve to form and maintain germinal cells, promote differentiation of naive B cells into memory B cells and plasma cells, and also to negatively select against auto-reactive B cells. Accumulating evidence has indicated the pivotal roles of T_FH_ cells in the generation of pathogenic autoantibodies and tissue injury in SLE. The frequency of CD4^+^CXCR5^+^PD1^+^ T_FH_ cells correlated with the number of plasma cells, anti-nuclear factor titer and disease activity in human SLE [85]. Furthermore, the number and percentage of CD4^+^CXCR5^+^PD1^+^ T_FH_ cells diminished following treatment with corticosteroids in SLE patients [85]. Nevertheless, not all studies in lupus patients agreed on the increase in circulating T_FH_. Wong *et al.* reported a decrease in peripheral blood T_FH_ cells although it was argued that such observation was due to a migration of these cells into the tissues [86]. The role of T_FH_ in the formation and maintenance of germinal centres was relevant to the pathogenesis of tubulo-interstitial inflammation in LN. Ectopic germinal centres as well as aggregates of T and B cells have been found within the tubulo-interstitial infiltrates of the renal biopsies obtained from LN patients, and the presence of these ectopic germinal centres and lymphoid aggregates are strongly associated with renal tubular basement membrane immune complex deposition [87]. Moreover, the centroblasts and plasmablasts within these intrarenal germinal centres and lymphoid aggregates were capable of clonal expansion and somatic hypermutation [87]. Th22, characterized by the production of IL-22, is another newly identified T helper subset that is found to be significantly increased in activity SLE patients [88]. Although preliminary data suggested that Th22 was increased in SLE patients with skin manifestations but decreased in patients with isolated nephritis, the role of Th22 in specific organ involvement remained undefined [89]. Th9, which characteristically secretes IL-9, has both pro-inflammatory and anti-inflammatory actions, but the overall role is more skewed to pro-inflammatory in autoimmune diseases [90]. Taken together, the data regarding Th9 and Th22 on SLE are still relatively limited and their roles in the pathogenesis of LN remain unclear.

## 5. Conclusions

Production of autoantibodies and aberrations of lymphocyte subsets are two important pathogenic mechanisms of developing renal diseases in SLE patients. In this context, anti-dsDNA stands out to be a very important autoantibody as the monitoring of its titre is widely adopted in clinical practice and shows good association with clinical disease activities. Moreover, ample works have demonstrated that anti-dsDNA can bind to different resident renal components and mediate immunological injury to the kidneys. Future researches would be worthwhile to elucidate the target antigen of anti-dsDNA on resident renal cells and also the downstream events following these anti-dsDNA-antigen binding. As for lymphocyte subsets, current evidence suggested that the B cell repertoire might assume more pivotal roles and data from recent clinical trials on biologics that target the B cell repertoire or associated cytokines have lent further support to this notion. While imbalance of T cell subsets have been implicated in local renal injury and the different histological manifestations of LN, the exact mechanism of interaction between various T cell subsets and the role Treg still remain undefined. A better understanding of these pathogenic mechanisms will help develop novel approaches for disease activity monitoring and therapy.
